# The Role of Chemotherapy in Patients With HER2-Negative Isolated Locoregional Recurrence of Breast Cancer: A Multicenter Retrospective Cohort Study

**DOI:** 10.3389/fonc.2021.653243

**Published:** 2021-03-05

**Authors:** Kyoungmin Lee, Sung Hoon Sim, Eun Joo Kang, Jae Hong Seo, Heejung Chae, Keun Seok Lee, Ji-Yeon Kim, Jin Seok Ahn, Young-Hyuck Im, Seri Park, Yeon Hee Park, In Hae Park

**Affiliations:** ^1^Division of Hemato-Oncology, Department of Internal Medicine, Korea University Guro Hospital, Korea University College of Medicine, Seoul, South Korea; ^2^Center for Breast Cancer, National Cancer Center, Goyang, South Korea; ^3^Division of Hematology-Oncology, Department of Medicine, Samsung Medical Center, Sungkyunkwan University School of Medicine, Seoul, South Korea; ^4^Department of Health Sciences and Technology, Samsung Advanced Institute for Health Science & Technology (SAIHST), Sungkyunkwan University, Seoul, South Korea

**Keywords:** breast cancer, isolated locoregional recurrence, molecular subtype, chemotherapy, disease free survival

## Abstract

**Background:** The role of chemotherapy for isolated locoregional recurrence (iLRR) of breast cancer has not been firmly established after local therapies.

**Methods:** We performed a multicenter, retrospective analysis to evaluate the clinical implications of chemotherapy in breast cancer patients with HER2-negative iLRR.

**Results:** Of a total of 277 patients, 146 (52.7%) received chemotherapy for iLRR. Median follow-up duration was 56.1 months. Eighty-six (31.0%) patients had luminal B-like and 100 (36.1%) had TNBC iLRR. There was a trend of longer disease free survival (DFS) in the chemotherapy group (4-year DFS: 70.4 vs. 59.5%, HR = 0.68, 95% CI 0.45–1.02, log-rank *p* = 0.059). When adjusted with clinically relevant factors, DFS was significantly prolonged with chemotherapy (adjusted HR = 0.61, 95% CI 0.40–0.94, *p* = 0.023). Subgroup analyses for DFS showed patients with disease free interval (DFI) <5 years or prior chemotherapy had a benefit from chemotherapy (adjusted HR = 0.57, *p* = 0.018; adjusted HR = 0.51, *p* = 0.005, respectively). Regarding the molecular subtypes, a longer DFS with chemotherapy was observed both in luminal B-like (4-year DFS: 77.8 vs. 55.0%, HR = 0.51, 95% CI 0.27–0.99, log-rank *p* = 0.048) and in TNBC patients (4-year DFS: 61.9 vs. 42.8%, HR = 0.49, 95% CI 0.24–1.02, log-rank *p* = 0.056), but not in luminal A-like.

**Conclusions:** The chemotherapy for iLRR of breast cancer should be individualized for each patient, considering DFI, prior chemotherapy, and molecular subtypes.

## Introduction

Early detection and advanced therapeutics using locoregional and systemic therapies have greatly improved the prognosis of early breast cancer (EBC). Even with proper treatment, some patients experience disease recurrences which are the leading cause of death. However, in patients with isolated locoregional recurrence (iLRR) without distant metastasis, both the opportunity for cure and the potential risk of subsequent distant metastasis may exist together (1, 2).

The main treatment strategy for iLRR of breast cancer is local therapy with curative surgical resection and/or radiotherapy (RT) (3); however, it is challenging to treat these patients because the survival of patients with iLRR markedly decreases with significant increases of metastasis within 2 years of salvage therapy (4, 5). Therefore, adjuvant hormonal therapy or anti-human epidermal growth factor receptor 2 (HER2) therapy is recommended as standard treatment for patients with hormone receptor positive or HER2-positive tumors following adequate local treatment for iLRR (4). On the other hand, there are no standard indications for applying adjuvant chemotherapy because its role in preventing progression of iLRR has not been firmly established (6). In the Chemotherapy as Adjuvant for Locally Recurrent breast cancer (CALOR) trial—the only randomized trial for iLRR, the authors showed the necessity of adjuvant chemotherapy in patients with completely resected iLRR, especially in estrogen receptor (ER)-negative breast cancer (7). It provided an important insight on the different effect of chemotherapy on ER positive or negative diseases, even though they were recurrent ones. However, the results of the CALOR study could not be a concrete evidence for the role of chemotherapy for iLRR because of the lack of statistical power due to relatively small sample size. In addition, it did not consider two distinct molecular subtypes in ER positive disease (luminal A and B) with different prognoses and responses to chemotherapy.

In this study, we addressed the impact of chemotherapy in breast cancer patients with HER2-negative iLRR using a real-world clinical data collected from tertiary academic hospitals, and explored which patients would benefit from adjuvant chemotherapy following local treatment for iLRR.

## Patients and Methods

### Study Population

We conducted a multicenter, retrospective analysis of breast cancer patients with HER2-negative iLRR. We searched institutional databases from three tertiary institutions in Republic of Korea: Korea University Guro Hospital (KUGH), National Cancer Center (NCC), and Samsung Medical Center (SMC) for patients diagnosed with EBC between January 2000 and December 2015, and with iLRR presenting as isolated first-failure events after mastectomy or breast conserving therapy. We extracted clinicopathologic variables for primary and recurrent tumors including patient demographics, tumor location, pathologic characteristics, and applied therapies. The chemotherapy applied following local treatment for iLRR was specified as salvage adjuvant chemotherapy. Data collection complied with the requirements of the local Institutional Review Board, and this study was conducted in accordance with the Declaration of Helsinki and the Guidelines for Good Clinical Practice.

iLRR was defined as reappearance of breast cancer in the region of the ipsilateral breast/chest wall (local recurrence) and/or recurrence involving regional lymph nodes considered resectable, including ipsilateral axillary, supraclavicular, infraclavicular, and internal mammary nodes (regional recurrence). The principle of local treatment for iLRR was complete surgical resection with negative margins (R0 resection), and if the patient had no history of RT on affected site, RT was added unless it did not overlap with the previous RT field. Molecular subtypes were retrieved from the pathologic report of the iLRR and classified as follows: luminal A-like [ER+ and/or PgR+, and Ki-67 <20% (KUGH and NCC) or <25% (SMC)], luminal B-like [ER+ and/or PgR+, HER2+, and Ki-67 ≥20% (KUGH and NCC) or ≥25% (SMC)], HER2-positive, and triple negative breast cancer (TNBC) (ER-, PgR-, and HER2-). In patients with absent or incomplete information at the time of recurrence, the molecular subtype of the iLRR was inferred from the information at the time of initial diagnosis.

We excluded patients with: (1) distant recurrence before iLRR; (2) contralateral breast recurrence; (3) no information on the molecular subtype at the time of recurrence and initial diagnosis; (4) HER2-positive disease; (5) no history of local treatment for iLRR; (6) or incomplete data including treatment records.

### Statistical Analyses

Qualitative or categorical variables were presented as frequency and proportion, and were compared using the chi-square test or Fisher's exact-test, as appropriate. Continuous variables were presented as median with range or 95% confidence interval (CI). For survival analysis, patients were followed from the diagnosis of iLRR until the last follow up or until death occurred, with the cut-off date of September 30, 2019. Disease-free survival (DFS) was calculated from the date of iLRR diagnosis to the date of treatment failure, including occurrence of another LLR, contralateral breast cancer, distant metastasis, or death from any cause. Overall survival (OS) was calculated from the date of diagnosis of iLRR to the date of death from any cause. Survival curves were estimated by the Kaplan-Meier method and compared using the log rank-test.

A multivariable Cox proportional hazard analysis was conducted to evaluate the impact of additional chemotherapy on DFS while adjusting covariates, including age at iLRR (≤50 or >50 years), disease free interval (DFI) from primary surgery to iLRR (≤5 or >5 years), history of previous chemotherapy for primary cancer (yes or no), location of recurrence (local or regional), and molecular subtype of recurred tumor. Similar analyses were conducted for subgroups under each variable, and presented in forest plots.

All statistical analyses were performed using IBM SPSS Statistics for Windows, Version 20.0 (IBM Corp., Armonk, NY, USA), and survival curves were generated using GraphPad Prism 5.0 (GraphPad Software, San Diego, CA, USA). A *p*-value of <0.05 was considered statistically significant.

## Results

### Patient's Characteristics

**Figure 1:**
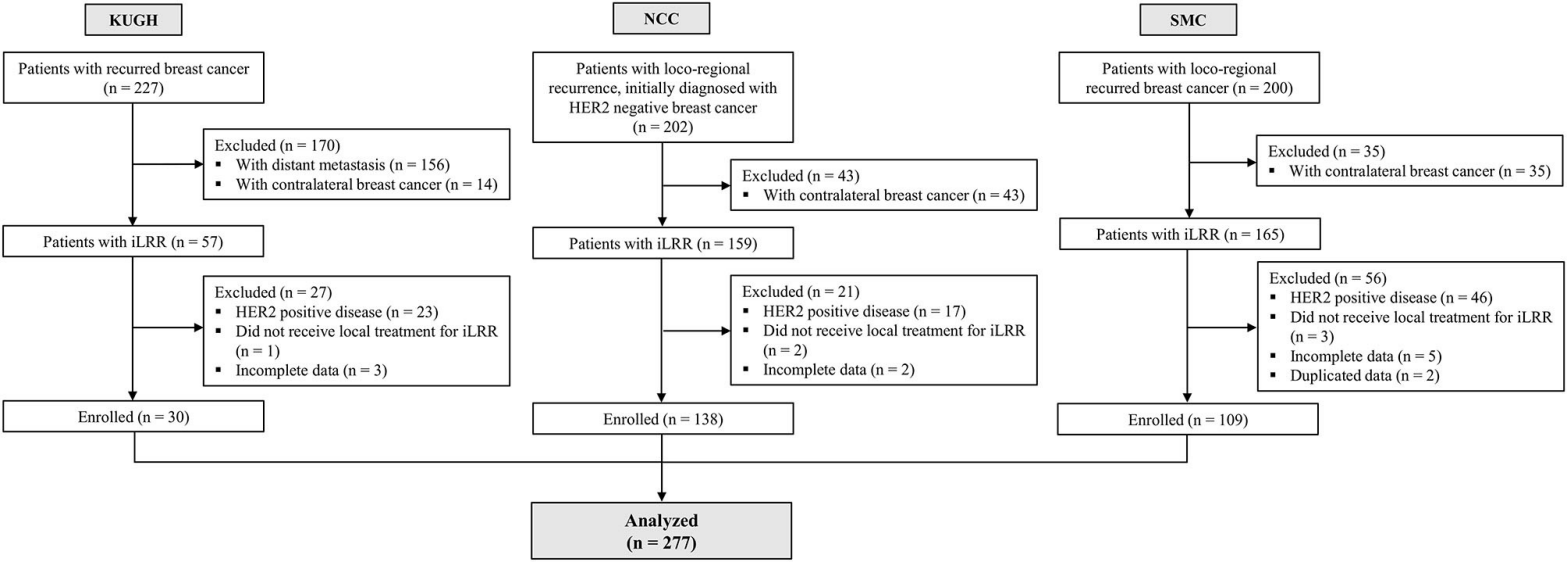
Summary of patient flow diagram.

We identified 277 patients with HER2-negative iLRR of breast cancer (30 from KUMC, 138 from NCC, and 109 from SMC) (Figure 1). About one third of these iLRR patients (*n* = 96, 34.7%) had regional recurrence, and the median age of patients at diagnosis of iLRR was 49 years (range, 25–79). The median DFI from primary diagnosis to iLRR was 37.2 months (range, 2.1–185.8), and 198 (71.5 %) patients had received adjuvant chemotherapy for their primary tumor, including 12 patients receiving neoadjuvant chemotherapy. Among the patients with history of prior adjuvant chemotherapy, most patients received anthracycline and/or taxane based regimens except for 11 patients treated with CMF (cyclophosphamide, methotrexate, and fluorouracil). The subtypes of recurred tumor consisted of 32.9% of luminal A-like (*n* = 91), 31.0% of luminal B-like (*n* = 86), and 36.1% of TNBC (*n* = 100). The missing histologies of 26 (9.4%) recurrent tumors were estimated from molecular information of their primary tumors.

Overall, 146 (52.7%) patients received chemotherapy after local therapies for iLRR (chemotherapy group). The chemotherapeutic regimens of salvage adjuvant chemotherapy were determined at the discretion of the physicians based on the history of previous chemotherapy, especially considering the use of anthracyclines or taxanes. Majority of patients (*n* = 117/146) received anthracycline and/or taxane based therapy as salvage adjuvant therapy. As a result, 97.3% of patients in the chemotherapy group (*n* = 142/146) received anthracycline and/or taxane based therapies at least once during the course of their treatment. There were no significant differences between those who received salvage chemotherapy for iLRR (chemotherapy group) and those who did not (no chemotherapy group), in terms of age at iLRR, DFI, prior chemotherapy, and location of recurrences. However, the distribution of molecular subtypes was quite different, with a high proportion of TNBC patients in the chemotherapy group, while a high proportion of luminal A-like patients in the no chemotherapy group. Further details were summarized in Table 1 and applied chemotherapy regimens in this study were presented in Supplementary Table 1.

**Table 1:** Baseline characteristics.

**Variables**	**All patients (*n* = 277)**	**Chemotherapy group (*n* = 146)**	**No chemotherapy group (*n* = 131)**	***p***
**Age at recurrence**				0.31
≤50 y	160 (57.8%)	89 (61.0%)	71 (54.2%)	
>50 y	117 (42.2%)	57 (39.0%)	60 (45.8%)	
**Interval from primary surgery to iLRR**				0.86
≤5 y	192 (69.3%)	100 (68.5%)	92 (70.2%)	
>5 y	85 (30.7%)	46 (31.5%)	39 (29.8%)	
**Type of primary surgery**				<0.01
Breast conserving surgery	194 (70.0%)	114 (78.1%)	80 (61.1%)	
Mastectomy	83 (30.0%)	32 (21.9%)	51 (38.9%)	
**Initial T stage**				0.15
T1	152 (54.9%)	82 (56.2%)	70 (53.4%)	
T2	113 (40.8%)	61 (41.8%)	52 (39.7%)	
T3–4	12 (4.3%)	3 (2.1%)	9 (6.9%)	
**Initial** ***N*** **stage**				0.35
N0	175 (63.2%)	98 (67.1%)	77 (58.8%)	
N1	76 (27.4%)	36 (24.7%)	40 (30.5%)	
N2–3	26 (9.4%)	12 (8.2%)	14 (10.7%)	
**Previous (neo)adjuvant chemotherapy**				0.27
No	79 (28.5%)	37 (25.3%)	42 (32.1%)	
Yes	198 (71.5%)	109 (74.7%)	89 (67.9%)	
**Location of recurrence**				1.00
Local	181 (65.3%)	95 (65.1%)	86 (65.6%)	
Regional^*^	96 (34.7%)	51 (34.9%)	45 (34.4%)	
**Molecular subtype of recurred tumor**				
Luminal A-like	91 (32.9%)	31 (21.2%)	60 (45.8%)	*<0.01*
Luminal B-like	86 (31.0%)	44 (30.1%)	42 (32.1%)	0.83
TNBC	100 (36.1%)	71 (48.6%)	29 (22.1%)	*<0.01*

*N, number; y, year*.

* *This included 18 patients with locoregional recurrence, 13 of whom received additional chemotherapy*.

### Survival Outcomes Following Treatment of iLRR

With a median follow up of 56.1 months (range, 0.5–161.8) after diagnosis of iLRR, 97 (35.0%) patients experienced breast cancer-related events after the completion of treatment for iLRR, and 52 (18.8%) patients died of breast cancer. The recurrence patterns were distant metastasis (*n* = 86, 88.7%), second locoregional recurrence (*n* = 9, 9.3%), and contralateral breast cancer (*n* = 1, 1.0%). The median DFS from iLRR among all 282 patients was 104.0 months (95% CI, 67.4–140.6), while the median OS was not reached. The 4-year DFS and OS rates were 65.6 and 83.9%, respectively.

**Figure 2:**
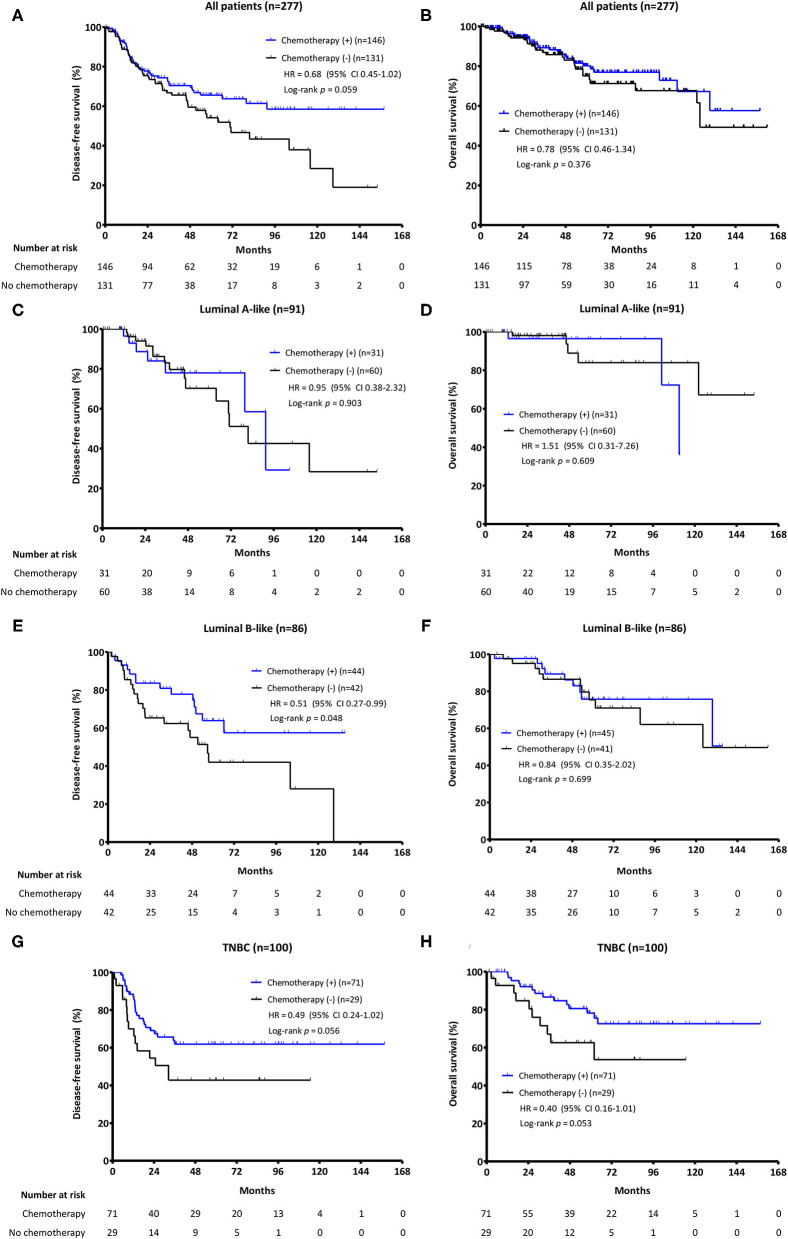
The Kaplan-Meier survival analysis for salvage adjuvant chemotherapy. PFS and OS according to the addition of chemotherapy **(A,B)** in all patients; **(C,D)** in Luminal A-like patients; **(E,F)** in Luminal B-like patients; and **(G,H)** in TNBC patients.

**Figure 3:**
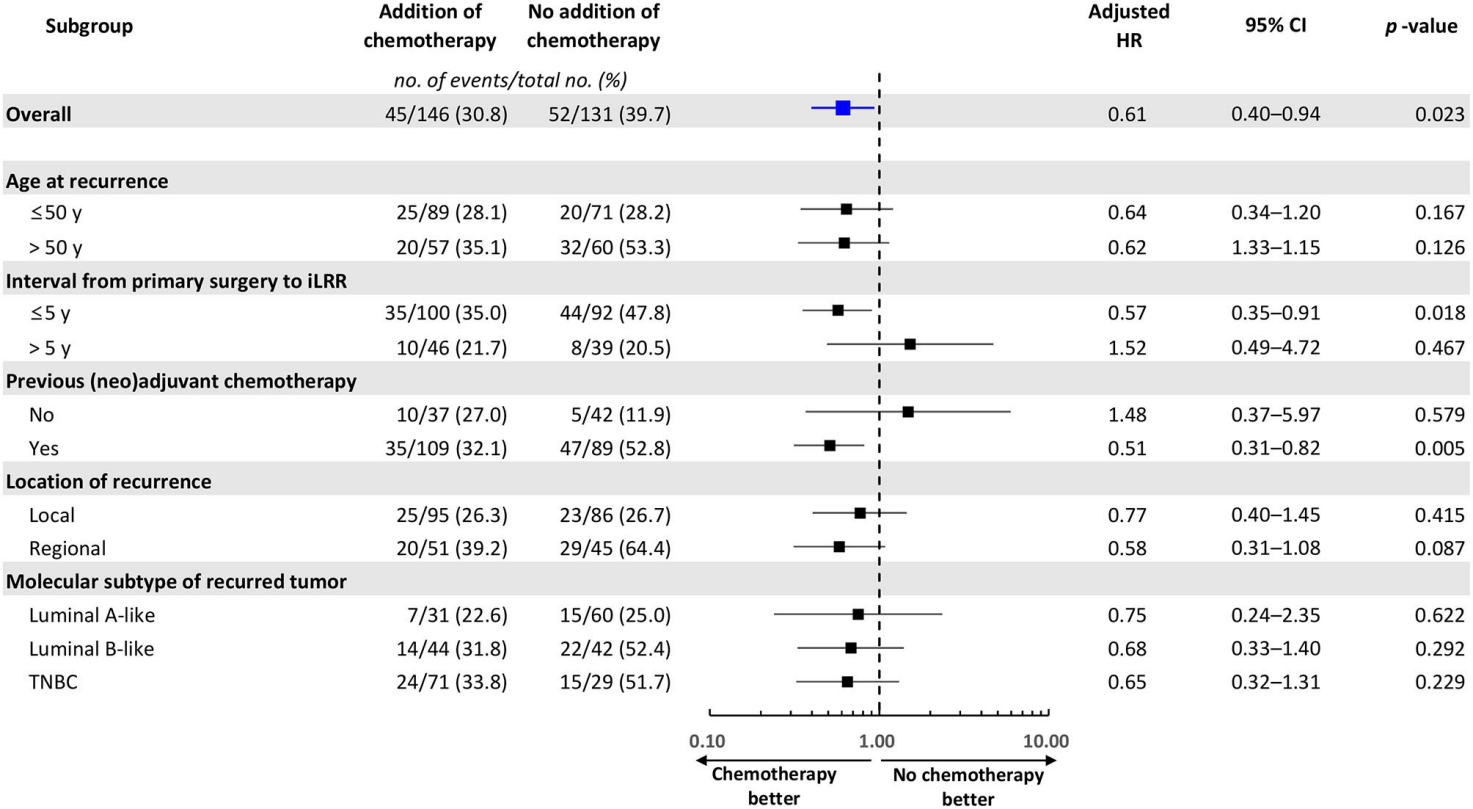
Forest plot of the impact of adjuvant chemotherapy for iLRR on disease-free survival in patient subgroups. Adjusted variables: age at iLRR (≤50 or >50 years), interval from primary surgery to iLRR (≤5 or >5 years), history of previous chemotherapy for primary cancer (yes or no), location of recurrence (local or regional), and molecular subtype of recurred tumor. No, number; iLRR, isolated locoregional recurrence; HR, hazard ratio; CI, confidence interval.

When the survival analysis was performed without any adjustment between two groups, there was a trend toward an improved DFS in the chemotherapy group (4-year DFS: 70.4 vs. 59.5%, HR = 0.68, 95% CI 0.45–1.02, log-rank *p* = 0.059), which was not observed for OS (4-year OS: 85.0 vs. 83.0%, HR = 0.78, 95% CI 0.46–1.34, log-rank *p* = 0.376) (Figures 2A,B). However, DFS was significantly prolonged with chemotherapy when adjusted with clinically relevant factors like age at iLRR, DFI, previous chemotherapy, location of iLRR, and molecular subtype (adjusted HR = 0.61, 95% CI 0.40–0.94, *p* = 0.023). The beneficial effect of chemotherapy on DFS was observed especially in patients with early recurrence (DFI <5 years) or previous (neo)adjuvant chemotherapy (adjusted HR = 0.57, 95% CI 0.35–0.91, *p* = 0.018; adjusted HR = 0.51, 95% CI 0.31–0.82, *p* = 0.005, respectively) (Figure 3).

### Analyses for the Benefit of Chemotherapy on iLRR by Molecular Subtypes

**Figure 4:**
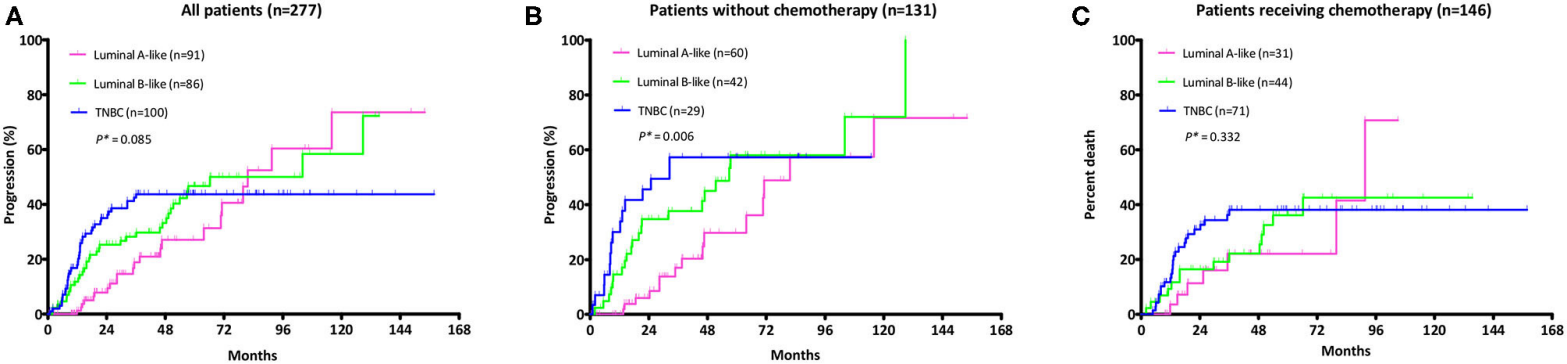
Cumulative recurrence rate after iLRR according to the subtype among **(A)** all patients; **(B)** patients did not receive chemotherapy for their iLRR; **(C)** patients who received chemotherapy for their iLRR. *Log rank *p* for trend.

Survival analysis by molecular subtypes showed a longer DFS with chemotherapy both in luminal B-like subtype (4-year DFS: 77.8 vs. 55.0%, HR = 0.51, 95% CI 0.27–0.99, log-rank *p* = 0.048) and in TNBC patients (4-year DFS: 61.9 vs. 42.8%, HR = 0.49, 95% CI 0.24–1.02, log-rank *p* = 0.056), but not in luminal A-like subtype. Additionally, there was a trend of OS extension in TNBC patients with chemotherapy (4-year OS: 80.5 vs. 62.6%, HR = 0.40, 95% CI 0.16–1.01, log-rank *p* = 0.053) (Figures 2C–H). Of note, the cumulative incidences of breast cancer recurrence after iLRR were significantly different by molecular subtypes. The cumulative incidence was continuously rising in luminal A-like patients regardless of chemotherapy. However, it reached a plateau at 60 months in luminal B-like patients with chemotherapy, which was not observed in patients without chemotherapy (Figure 4).

## Discussion

Breast cancer is considered a heterogeneous disease entity comprised of four and more subtypes (8). Primary systemic treatment is recommended based on molecular subtypes and clinical prognostic factors such as tumor size, nodal metastasis, age, and proliferative index (9–11). The incidence of iLRR is decreasing with improvements in diagnostic and therapeutic management of primary disease from ~1% per year to 0.5% per year (12). However, our study shows that distant metastases occurred in about 30% of patients with iLRR, which implies that iLRR could be a preceding event to distant metastases. Therefore, more integrative and multidisciplinary approaches considering recurrence sites, tumor burden, prior treatment, and patient preference are needed to prevent a second recurrence.

Clinical trials with systemic chemotherapy in patients with iLRR are limited due to relatively low incidence and heterogeneous disease entities. The CALOR trial was the only prospective randomized study exploring the role of systemic chemotherapy in patients with completely excised iLRR, though it failed to include all planned number of patients. In that trial, adjuvant chemotherapy improved the 5-year DFS in patients with ER-negative disease (*n* = 58), HR of 0.32 (7). Moreover, the benefit of chemotherapy in patients with ER-negative iLRR was maintained even after 10 years of follow-up. Meanwhile, no benefit from chemotherapy was detected in ER positive group (13).

To confirm the role of chemotherapy in iLRR with different molecular subtypes, we performed real world data analysis of patient population with the same conditions as the CALOR trial. Based on our results, we observed a trend of improving DFS by adding adjuvant chemotherapy for iLRR of breast cancer. Considering heterogeneous patient population, disease free survival analysis adjusted with relevant clinical factors was conducted including age, DFI, prior treatment, and recurrence site. Adjusted survival analysis revealed that chemotherapy significantly prolonged DFS in the overall population. In subgroup analysis, the benefits of chemotherapy were more pronounced in patients with shorter DFI from primary surgery to iLRR and history of previous chemotherapy for primary cancer.

In our study, most TNBC patients (*n* = 71, 71.0%) received salvage adjuvant chemotherapy while some did not due to small tumor size (<10 mm) or patient refusal. Such imbalance in patient characteristics may have been the reason for the failure to statistically prove the benefit of chemotherapy in this high risk group. The luminal B-like group was also expected to have benefit from chemotherapy based on previous studies suggesting that luminal B subtype breast cancers exhibited more aggressive clinical features than luminal A subtype, while the benefits of chemotherapy were higher (14–16). In our study, 51.2% of patients with luminal B-like disease received chemotherapy. The cumulative recurrence rate increased without chemotherapy use in both the luminal A and B-like population, which was a typical pattern of the luminal subtype (17–19). However, it plateaued in the luminal B-like group with chemotherapy similar to that of TNBC group. According to our results, it would be reasonable to consider chemotherapy followed by endocrine therapy in patients with luminal B-like iLRR, especially those having a short disease-free interval <5 years or prior chemotherapy for primary tumor.

Based on the retrospective nature of our study, there are several limitations. First, we conducted molecular subtyping according to the pathologic results of each hospital. Although breast cancer can be divided into several intrinsic molecular subtypes according to the gene expression profiles, immuno-histochemistry based molecular subtyping is still mainly used in general clinical practice (20, 21). However, the degree of consistency of test results among institutions can always be an important issue. In our study, two hospitals reported Ki-67 as continuous value and the other used categorical value. Therefore, we used two cut-off values to divide Ki-67; 20% for continuous values and 25% for categorical values. Second, we could not analyze the interaction between different chemotherapeutic agents and their clinical efficacies. Chemotherapeutic regimens for the salvage adjuvant chemotherapy were so heterogeneous as it was largely determined by clinical factors such as previous treatment, patient preferences, or co-morbidity. Last, performance status (PS), an important prognostic factor, was not included in this retrospective study. PS is an important factor in deciding whether to take chemotherapy, so the lack of data on PS could be one of the drawbacks of this study.

In conclusion, additional chemotherapy for iLRR of breast cancer should be individualized for each patient, especially considering molecular subtypes of recurrent tumors, disease free interval between primary cancer and iLRR, and previous adjuvant chemotherapy. Our qualified real world data would be a good supporting evidence to establish an appropriate treatment for iLRR in HER2 negative breast cancer.

## Data Availability Statement

The original contributions generated in the study are included in the article/Supplementary Material, further inquiries can be directed to the corresponding authors.

## Ethics Statement

The studies involving human participants were reviewed and approved by the Institutional Review Board of each institution (IRB Approval Number: 2020GR0088 for KUGH, NCC2019-0302 for NCC, 2020-04-205 for SMC). Written informed consent for participation was not required for this study in accordance with the national legislation and the institutional requirements.

## Author Contributions

KL: methodology, formal analysis, investigation, writing—original draft, and funding acquisition. SS: conceptualization, investigation, resources, and project administration. EK, JS, HC, KL, J-YK, JA, and Y-HI: validation, resources, and supervision. SP: data curation, and resources. YP: conceptualization, validation, investigation, supervision, and project administration. IP: conceptualization, methodology, writing—review and editing, project administration, and funding acquisition. All authors have read and approved the manuscript and IP took responsibility for submitting the manuscript for publication.

## Conflict of Interest

The authors declare that the research was conducted in the absence of any commercial or financial relationships that could be construed as a potential conflict of interest.
