# Supplementary material for: The Role of Chemotherapy in Patients With HER2-Negative Isolated Locoregional Recurrence of Breast Cancer: A Multicenter Retrospective Cohort Study
Source: Front Oncol. 2021 Mar 5;11:653243. doi: 10.3389/fonc.2021.653243 (PMC7973367; doi:10.3389/fonc.2021.653243)
Supplement: Supplementary file 1 [file Table_1.DOCX]

Supplementary Material

# Supplementary Table 1. Chemotherapy regimens used in this study

| **Variables** | **Chemotherapy group**  **(n = 146)** | **No chemotherapy group**  **(n = 131)** |
| --- | --- | --- |
| **Prior adjuvant chemotherapy for primary tumor** | 109 (74.7%) | 89 (67.9%) |
| Anthracycline and cyclophosphamide | 23 | 19 |
| Fluorouracil, anthracycline, and cyclophosphamide | 44 | 25 |
| Cyclophosphomide, methotrexate, and fluorouracil | 6 | 5 |
| Anthracycline and cyclophosphamide followed by taxane | 34 | 35 |
| Anthracycline and taxane | 1 | 2 |
| Docetaxel, doxorubicin and cyclophosphamide | 0 | 1 |
| Docetaxel and cyclophosphamide | 0 | 1 |
| Taxane monotherapy | 1 | 1 |
| **Salvage adjuvant chemotherapy for iLRR** |  |  |
| Anthracycline and cyclophosphamide | 8 |  |
| Fluorouracil, anthracycline, and cyclophosphamide | 10 |  |
| Cyclophosphomide, methotrexate, and fluorouracil | 18 |  |
| Anthracycline and cyclophosphamide followed by taxane | 20 |  |
| Anthracycline and taxane | 2 |  |
| Epirubicin and cisplatin | 1 |  |
| Docetaxel and cyclophosphamide | 34 |  |
| Taxane and cisplatin | 10 |  |
| Taxane monotherapy | 32 |  |
| Gemcitabine and cisplatin | 3 |  |
| Capecitabine and cisplatin | 1 |  |
| Capecitabine monotherapy | 2 |  |
| Others/unknown | 5 |  |
